# Differential expression of receptors mediating receptor-mediated transcytosis (RMT) in brain microvessels, brain parenchyma and peripheral tissues of the mouse and the human

**DOI:** 10.1186/s12987-020-00209-0

**Published:** 2020-07-22

**Authors:** Wandong Zhang, Qing Yan Liu, Arsalan S. Haqqani, Sonia Leclerc, Ziying Liu, François Fauteux, Ewa Baumann, Christie E. Delaney, Dao Ly, Alexandra T. Star, Eric Brunette, Caroline Sodja, Melissa Hewitt, Jagdeep K. Sandhu, Danica B. Stanimirovic

**Affiliations:** 1 Human Health Therapeutics Research Centre, National Research Council of Canada, 1200 Montreal Road, M54, Ottawa, ON K1A0R6 Canada; 2 Scientific Data Mining/Digital Technology Research Centre, National Research Council of Canada, Ottawa, Canada

**Keywords:** Blood–brain barrier, Receptor-mediated transcytosis, Isolated brain microvessels, RNAseq, Mouse and human species, IGF1R, Transferrin receptor

## Abstract

Receptor-mediated transcytosis (RMT) is a principal pathway for transport of macromolecules essential for brain function across the blood–brain barrier (BBB). Antibodies or peptide ligands which bind RMT receptors are often co-opted for brain delivery of biotherapeutics. Constitutively recycling transferrin receptor (TfR) is a prototype receptor utilized to shuttle therapeutic cargos across the BBB. Several other BBB-expressed receptors have been shown to mediate transcytosis of antibodies or protein ligands including insulin receptor (INSR) and insulin-like growth factor-1 receptor (IGF1R), lipid transporters LRP1, LDLR, LRP8 and TMEM30A, solute carrier family transporter SLC3A2/CD98hc and leptin receptor (LEPR). In this study, we analyzed expression patterns of genes encoding RMT receptors in isolated brain microvessels, brain parenchyma and peripheral organs of the mouse and the human using RNA-seq approach. IGF1R, INSR and LRP8 were highly enriched in mouse brain microvessels compared to peripheral tissues. In human brain microvessels only INSR was enriched compared to either the brain or the lung. The expression levels of SLC2A1, LRP1, IGF1R, LRP8 and TFRC were significantly higher in the mouse compared to human brain microvessels. The protein expression of these receptors analyzed by Western blot and immunofluorescent staining of the brain microvessels correlated with their transcript abundance. This study provides a molecular transcriptomics map of key RMT receptors in mouse and human brain microvessels and peripheral tissues, important to translational studies of biodistribution, efficacy and safety of antibodies developed against these receptors.

## Background

The receptor-mediated transcytosis (RMT) is a vesicular transcellular route by which various macromolecules are transported across a barrier, typically formed by a cell monolayer [1, 2]. This process is particularly important for brain delivery of essential macromolecules, including transferrin and insulin [3–6], across the blood–brain barrier (BBB) [1, 2]. A ligand binding to a receptor on the luminal surface of brain endothelial cells (BEC) triggers ligand-receptor complex endocytosis, routing through various intracellular endosomal compartments where cargo is detached from the receptor and released on the abluminal side, while the receptor recycles ‘back’ to accept additional cargo molecules [7–11]. This pathway has been particularly well described for the transferrin receptor (TfR), which undergoes constitutive recycling and has been considered a ‘prototypical’ trigger of the RMT pathway [7–13].

Antibodies and peptide ligands against various endocytosing BBB receptors have been developed as potential molecular Trojan horses or shuttles to deliver therapeutic cargos across the BBB [2, 8–16]. Discovery strategies utilizing molecular (‘omics’) analyses of the BBB and screening of antibody libraries to select antibody species that can transmigrate the BBB [17–22] have yielded an experimental proof of concept for novel RMT-antibody pairs. The literature survey of BBB targets shown to mediate transcytosis of antibodies or protein ligands in various model systems in vitro and in vivo, identified the following broad classes of transporters: (a) *iron transporters*: transferrin receptor (TFRC) [3–6, 8, 12, 13]; (b) *insulin and insulin*-*like growth factors receptors*: INSR, IGF1R, and IGF2R [16, 23, 24]; (c) *lipid transporters*: low-density lipoprotein receptor (LDLR), LDLR-related protein 1 (LRP1), LRP8, and transmembrane protein 30A (TMEM30A/CDC50A) [25–32]; (d) *solute carrier family transporters*: glucose transporter-1 (GLUT-1)/SLC2A1 and SLC3A2/CD98hc [33, 34]; and e) *neuropeptide receptors*: leptin receptor (LEPR) [35]. Structure/function relationships, ligand interactions and signaling, and specialized biology of these receptors is vastly different (summarized briefly in Additional file 1: Table S1), expanding the spectrum of potential RMT pathways across the BBB beyond the ‘canonical’ mechanism of TfR recycling. Whereas the mechanisms of antibody transport by some of these targets, in particular SLC family of transporters, is not fully understood, for the purpose of this manuscript they will be considered in comparison with ‘classical’ RMT-triggered pathways.

The development of peptides and antibodies that bind RMT receptors as potential delivery carriers for brain-targeting biotherapeutics brought about further understanding of RMT mechanisms and underscored key limitations of various approaches. For example, evaluation of bi-specific antibodies where the TfR antibody was used as a BBB transport shuttle [36, 37] revealed TfR antibody-driven fast systemic clearance, on-target complement-mediated toxicity on TfR-rich reticulocytes, as well as species differences in transport capacity in mice and non-human primates [7, 36, 38]. Ideally, RMT receptors targeted for the development of BBB delivery antibodies should demonstrate a high degree of BBB expression selectivity compared to peripheral organs, a high abundance in the brain endothelium, as well as low expression in the brain parenchyma [8, 9]. This profile would enable an extended systemic half-life and reduced toxicity, high efficiency of brain delivery and low target-mediated removal in the brain tissue. For translational preclinical studies, and, ultimately, for demonstration of the concept in human patients, it is also important to consider species differences in the RMT target expression [39]. Three BBB carriers targeting RMT pathway, a peptide ligand of LRP-1 (Angiopep) [26, 27, 40], anti-human INSR antibody [14, 15, 41], and anti-TfR antibody [42] are being tested in clinical trials; however, the clinical proof of concept for RMT pathway as a viable route for systemic delivery of CNS biotherapeutics remains inconclusive.

The principal objective of the current study was to compare the transcript abundance of selected receptors and transporters previously shown to mediate transport of antibodies or protein ligands into the brain in isolated brain microvessels from mouse, the principal experimental species used in pre-clinical studies, and from human, by using RNA-seq. In addition, transcript abundance for these receptors was determined in the whole brain tissue and in various peripheral organs. Significant species differences in RMT receptor abundances and distribution among brain microvessels, brain and peripheral organs were observed between mouse and human. This study suggests that the RMT target receptor abundance is an important parameter to consider in translational studies for antibody-based BBB delivery strategies.

## Materials and methods

### Human and mouse tissues

Samples of the human brain and lung tissues from three individuals were used in the study. Human lung tissues were from normal adjacent lung tissues of three non- small cell lung cancer (NSCLC) patients undergoing surgical resections. The surgical samples were deposited in the Lung Tumor Bank managed by the CDHA-Capital District Health Authority at Halifax, NS, Canada. All patients signed informed consent as per CDHA-RS/2013-271, which allowed their tissues to be archived in the CDHA Lung Tumor Bank for molecular studies. Human post mortem brain tissues (two females and one male, 73–76 years old; deceased from non-brain related pathologies) were obtained from the Human Brain and Spinal Fluid Resource Center, VAMC (Los Angeles, CA), which is sponsored by NINDS/NIMN, National Multiple Sclerosis Society, VA Greater Los Angeles Healthcare System, and Veterans Health Services and Research Administration, Department of Veteran Affairs. The use of human tissues in this study was approved by the Research Ethics Board of the National Research Council Canada (Protocols #2013-38 and #2006-03). The use of mice in this study was approved by the Animal Care Committee of the Human Health Therapeutics Research Centre at the National Research Council of Canada (Animal User Protocol #2016-04). Mice (C57BL/6 J strain, three males, 9-month old) were perfused transcardially with heparnized saline (15 mL at 2 mL/min) and organs were collected. Brain and brain vessels were freshly isolated without freezing; other organs were frozen at − 80 °C until RNA isolation.

### Isolation of microvessels and capillaries from tissues

Microvessels and capillaries were isolated from mouse and human brains, as well as mouse lungs using modified protocols described previously [43]. Mouse brain and lung vessels were isolated from fresh tissues, whereas all human tissues were frozen and stored at − 80 °C until vessel isolation. The tissues were pre-weighed and thawed briefly at room temperature. Tissue homogenization and vessel separation were performed on ice using PBS (Wisent, St-Bruno, QC) containing protease inhibitor cocktail (Sigma Aldrich, St. Louis, MO.) or using a buffer containing 150 mM NaCl, 50 mM Tris (BDH, Ward Chester, PA) pH 8.0 for mass spectrometry analysis with instruments pre-chilled on ice. Respective tissues were chopped using razor blade, placed in a 5-mL Wheaton Dounce homogenization tube (Fisher Scientific, Hampton, NH), and 5 mL of PBS buffer was added per tube. Tissue was homogenized with 10 strokes of the pestle connected to Eberbach Con-Torque Homogenizer (Fisher Scientific, Hampton, NH). The tissue homogenate was transferred onto pluriStrainers in a 50-mL conical tube with a connector ring and strainers (pluriSelect, San Diego, CA) in descending order, as follows: 300 µm, 100 µm, and 20 µm strainers and connector ring, and then gentle suction was used to filter the homogenate through strainers.

Stacked strainers were rinsed with 5–10 mL of PBS and vessels were collected by placing strainers upside down in a new 50-mL conical tube followed by a rinse with 5-mL PBS per each strainer. To release the microvessels and capillaries from the strainer, the buffer was forced through the filter by pipetting up and down with a P1000 pipette. Vessels collected onto 100 μm and 20 μm strainers were collected in the same 50-mL conical tube and were centrifuged at 900*g* for 5 min at 4 °C to pellet the vessels. The supernatant was carefully aspirated, and vascular pellets, designated as brain microvessels (BMV) were then processed for proteomics, immunofluorescence and RNA extraction. Vessel-depleted brain filtrates were also collected to analyze protein expression in brain parenchyma.

### RNA isolation

RNA was extracted from isolated BMVs by using RNeasy Plus Mini kit (Qiagen, Toronto, ON), while NucleoSpin RNA plus kit (Macherey–Nagel GmbH & Co. KG) was used for RNA isolation from all other tissues following manufacturer’s protocols. Genomic DNA contamination was removed by Turbo DNA-Free Kit (Life Technologies/ThermoFisher Scientific, Nepean, ON). RNA quality was assessed using Agilent Bioanalyzer 2100 (Santa Clara, CA).

### RNA-seq

RNA-Seq Libraries were generated using the TruSeq strand RNA kit (Illumina, San Diego, CA). The RNA-Seq libraries were quantified by Qbit and qPCR according to the Illumina Sequencing Library qPCR Quantification Guide and the quality of the libraries was evaluated on Agilent Bioanalyzer 2100 using the Agilent DNA-100 chip (Santa Clara, CA). The RNA-Seq library sequencing was performed using Illumina Next-Seq 500. FASTQ file format was processed by trimming the adaptor sequences, filtering low-quality reads (Phred Score ≤ 20) and eliminating short reads (length ≤ 20 bps) using software package FASTX-toolkit [http://hannonlab.cshl.edu/fastx_toolkit/]. STAR (v2.5.3a) [44] was used for the alignment of reads to the reference genome and to generate gene-level read counts. RSEM (version 1.3.3) [45] was used for alignment, to generate Transcripts per million (TPM) count. Mouse reference genome (version GRCm38.p6, M24), human reference genome (version GRCh38.p13, Genecode 33) and corresponding annotations were used as references for RNA-seq data alignment process. DESeq 2 [46] was used for data normalization and differentially expressed gene identification for each pair-wise comparison.

### Public data sets and analysis

RNA-seq and microarray data in the public domains were obtained to compare/benchmark the data generated from this study for quality and comparability purposes. For RNA-seq data, raw data corresponding to normal lung and brain samples were obtained from the Sequence Read Archive [47] from the Genomics Data Commons [48]. GTEx data were processed using GDC reference files using GDC mRNA analysis pipeline (STAR two-pass) [44]. These data were combined with 12 samples analyzed at NRC and processed using DESeq 2 [46].

### Automated Western blot analysis (Wes™)

Human and mouse BMVs were lysed in Cellytic MT buffer (Sigma) with 1 X Complete protease inhibitor (Roche) pellets on ice. The lysates were incubated on ice for 30 min, vortexed, then centrifuged at 21,000×*g* for 10 min in a Sorvall Legend Micro 21R centrifuge. Protein concentrations were determined using the Quantipro BCA Assay Kit (Sigma). Wes was run using the 12–230kDA separation module (ProteinSimple), and the mouse or rabbit detection module (ProteinSimple Inc., San Jose, CA). Wes samples (protein at 0.8 mg/mL) were prepared by combining Master Mix to sample in a 1:4 ratio. Samples and Biotinylated Ladder were heated in a Accublock digital dry bath at 95 °C for 5 min. Samples were cooled to room temperature, vortexed to mix and centrifuged in a Mandel mini microfuge. Biotinylated ladder, samples, primary and secondary antibodies, and luminol were loaded on the plate and Wes was run using the standard protocol. Primary antibodies were rabbit anti-IGF1 receptor β (Cell Signaling, 3027S), mouse anti-transferrin receptor (Invitrogen, 13-6800) and rabbit anti-LRP1 (Abcam, ab925443). Primary antibodies were cross-reactive with human and mouse IGF1R, LRP1 and TfR proteins. Streptavidin-HRP was used to detect the ladder proteins. Data for each sample was first normalized to β-actin in the same lane. The level of the protein in mouse BMV was set as one fold. The fold-change of human protein was calculated relative to mouse protein (Mean ± SD).

### Immunofluorescence

Isolated brain microvessels in PBS (5 µL) were deposited on Superfrost Plus slides (Fisher Scientific, Toronto, ON) and air-dried for 30 min. Samples were then fixed in Methanol (Fisher Scientific, Toronto, ON) for 15 min at room temperature and followed by incubation in 5% Normal Goat or Normal Donkey Serum (Jackson ImmunoResearch, West Grove, PA) in 0.2% Triton X-100 (Sigma, Oakville, ON) solution for 1 h at room temperature. The vessels were incubated with CD31 antibody 1:300 (BD Biosciences, San Jose, CA), Collagen IV antibody 1:300 (Millipore, Etobicoke, ON) or Griffonia Simplicifolia Lectin I (GSL-I) 1:500 (Vector Labs, Burlingame, CA). The primary antibodies for TfR, LRP-1 and IGF1R are the same as used above for Wes™ analyses. Secondary antibodies used were Goat anti-Rat and Donkey anti-Goat Alexa conjugated (Fisher Scientific, Toronto, ON) at 1:500 dilutions for 1 h at room temperature. Samples were mounted in Fluorescent Mounting Medium (Dako, Santa Clara, CA) Containing 2 µg/mL Hoechst (Fisher Scientific, Toronto, ON). Images were acquired using Zeiss Axiovert 200 M microscope with 20× objective.

### Tissue-Immunocytochemistry

Isolated brain vessels were air-dried for 15 min on slide warmer at 37 °C and fixed using commercially available Zinc 10× fixative (diluted to 1x with MQH_2_O) for 90 min at RT. The slides were rinsed 3 times with PBS containing 0.05% tween-20 and blocked with Dako serum-free protein block containing 0.1% Triton-X 100 for 1 h at room temperature (RT). Endogenous peroxidase was quenched with 3% H_2_O_2_ in PBS for 15 min RT. The slides were rinsed 3 times with PBS containing 0.05% tween-20. IGF1R antibody (at 1:100 dilution; Cell Signaling 3027S lot13) was applied to the slides for 2 h RT (diluted in Dako antibody diluent containing 0.05% Triton-X 100). The slides were washed 3 times with PBS containing 0.05% tween-20 (at 2 min each). Secondary biotinylated goat anti-rabbit antibody was then applied to the slides for 45 min RT (1:250, diluted in Dako antibody diluent). The slides were washed 3 times with PBS containing 0.05% tween-20 (at 2 min each). RTU Horseradish peroxidase Avidin D was then applied for 30 min at RT. The slides were washed 3 times with PBS containing 0.05% tween-20 (at 2 min each). Color was developed 8 min with DAB substrate kit Vector labs SK4100. Slides were counterstained using hematoxylin, then dehydrated, cleared and cover slipped with xylene-based mounting media. The slides were visualized by Olympus IX81 inverted microscopy (Richmond Hill, ON). Images were acquired using Olympus IX81 inverted microscopy with 20× objective.

### Nano LC–MS/MS analysis

Peptides from digested proteins prepared as described [10] were analyzed on a reversed-phase nanoAcquity UPLC (Waters, Milford, MA) coupled with a LTQ-XL mass spectrometer (ThermoFisher, Waltham, MA) with a nano-electrospray interface operated in positive ion mode. The analysis involved injection and loading of 0.6–1 μg of mouse brain protein onto a 300 µm I.D. × 0.5 mm 3 µm PepMaps^®^ C18 trap (ThermoFisher) followed by eluting onto a 100 µm I.D. × 10 cm 1.7 µm BEH130C18 nanoLC column (Waters). The mobile phase consisted of 0.1% (v/v) formic acid in water as buffer A and 0.1% (v/v) formic acid in acetonitrile as buffer B. The peptides were separated using a gradient ramping from 0.1 to 45% buffer B over 40 min, 45%–85% buffer B over 2 min, and then re-equilibrating from 85 to 0.1% buffer B over 10 min at a flow rate of 600 nL/min. The eluted peptides were ionized by electrospray ionization (ESI). Data were acquired on ions with mass/charge (m/z) values between 350 and 2000 followed by three data-dependent MS/MS scans using collision-induced dissociation (CID) for fragmentation of the peptide ions.

### Statistical analysis

The data from multiple groups were analyzed and compared using One-way ANOVA followed by Tukey’s multiple comparisons test. Unpaired two-tailed student *t* test was used to compare data between two groups. p < 0.05 is considered statistical significance.

## Results

### Characterization of isolated brain microvessels (BMVs)

**Fig. 1:**
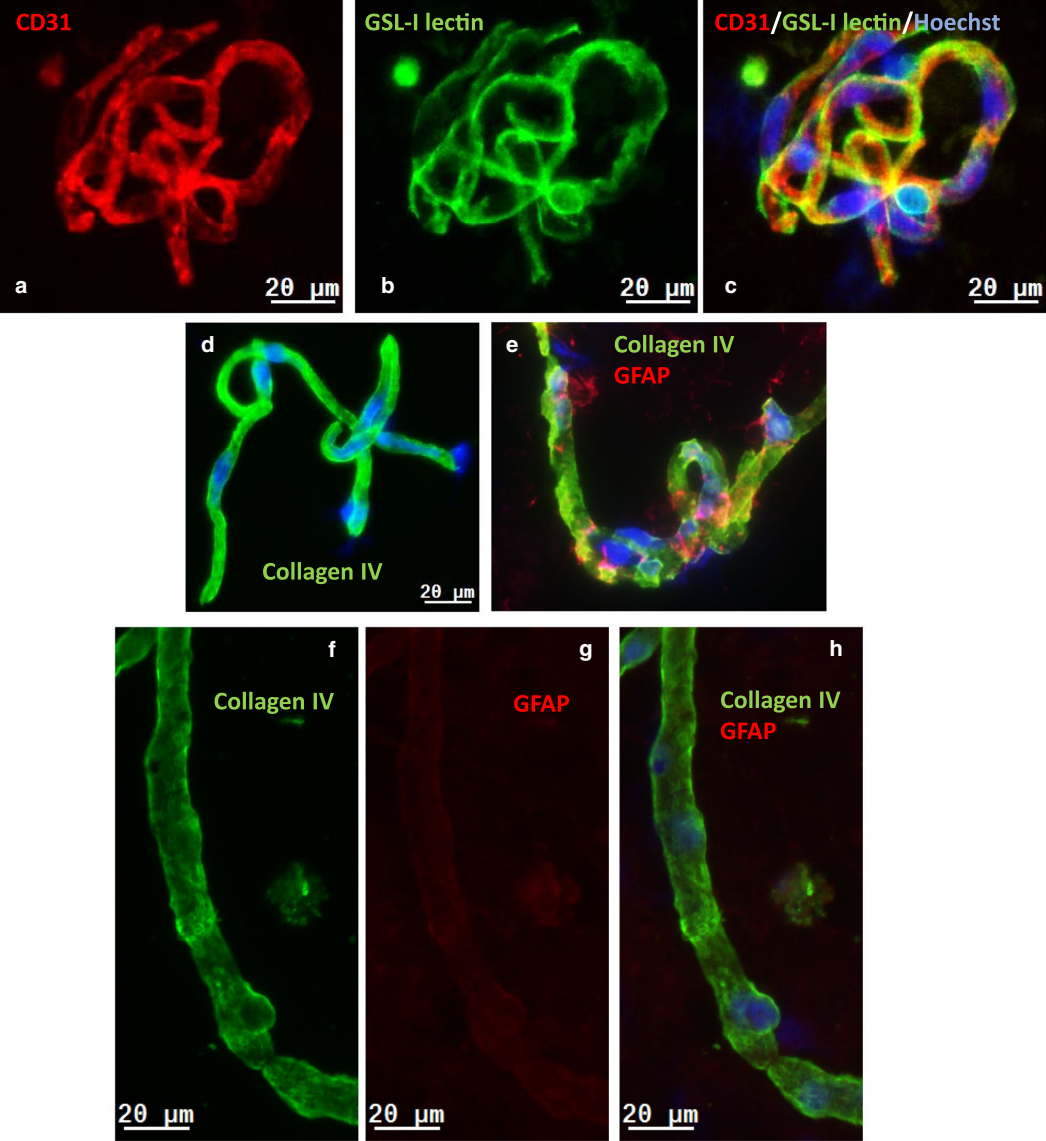
Immunofluorescence analysis of isolated brain microvessels and capillaries (BMV) from the mouse (**a–e**) and human (**f–i**) brain. **a** CD31 immunofluorescence (red) in mouse BMV; **b** GSL-I lectin staining (green) in mouse BMV. **c** A composite image of CD31, GSL-I and Hoechst-stained nuclei (blue) of the same BMV. **d** Collagen IV immunofluorescence (green) in mouse BMV. **e** A composite image of collagen IV (green) and GFAP (red) immunofluorescence and Hoechst-stained nuclei (blue) in mouse BMV. **f** Collagen IV immunofluorescence of human BMV. **g** GFAP immunofluorescence of human BMV. **h** A composite image of of collagen IV (green) and GFAP (red) immunofluorescence and f Hoechst-stained nuclei (blue) in human BMV. **i** Hoechst staining of nuclei in human BMV. The scale bar is 20 µm

Mouse (Fig. 1a–e) and human (Fig. 1f–i) BMVs isolated using a modified separation protocols described [43] were characterized by immunofluorescence staining for the endothelial antigen CD31/PECAM-1, the basement membrane component collagen IV and the astrocyte (end-feet) marker glial fibrillary acidic protein (GFAP). The luminal surface of endothelial cells was visualized using lectin GSL-1 (Fig. 1b), which binds mouse endothelial glycocalyx. BMVs exhibited a strong immunoreactivity for CD31/PECAM-1 (Fig. 1a, c), continuous staining with the abluminal marker collagen IV, as well as a weak and ‘spotty’ immunoreactivity for GFAP (Fig. 1e, g). The majority of vessels in analyzed samples measured less than 20 μm in diameter.

**Fig. 2:**
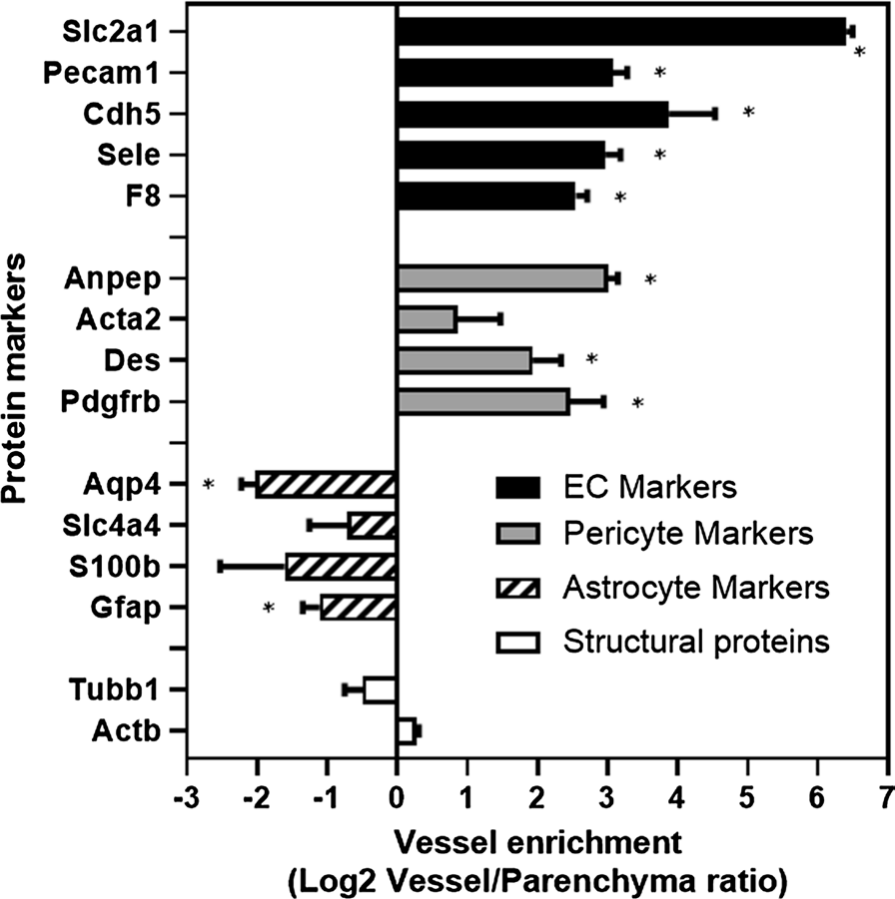
The enrichment of the blood–brain barrier (BBB)-related proteins in mouse brain microvessels (BMV) compared to brain parenchyma. BMVs and vessel-free brain parenchyma were isolated as described in Materials and Methods and relative enrichment of key protein markers in each fractions was determined by proteomics. Shown are Log2 ratios between levels of various proteins in purified vessels and vessel-depleted parenchyma. Protein markers correspond to: (1) *Endothelial cell markers*: Slc2a1, Glucose transporter (Glut1); F8, Coagulation factor VIII-related antigen; Sele, E-selectin; Cdh5, VE-cadherin; Pecam1, CD31; (2) *Pericyte markers*: Pdgfrb, Platelet-derived growth factor receptor beta; Des, Desmin; Acta2, smooth muscle actin; Anpep: CD13 pericyte marker (Aminopeptidase N); (3) *Astrocyte markers*: Gfap, Glial fibrillary acidic protein; S100β, Protein S100-beta; Slc4a4, Electrogenic sodium bicarbonate cotransporter 1; Gja1, Gap junction alpha-1 protein; Aqp4, Aquaporin-4; (4) *Structural proteins*: Actb, Cytoplasmic actin; Tubb1, beta tubulin. Error bars represent mean ± SD of three biological replicates

The enrichment of cell-specific markers in BMVs in comparison to *vessel*-*depleted* brain parenchyma, was also analyzed using targeted nanoLC-MS/MS. A relative BMV enrichment of specified proteins was presented in Log2 ratio in Fig. 2. *Endothelial cell markers* coagulation factor VIII-related antigen (F8), E-selectin (Sele), VE-cadherin (Cdh5) and Pecam1/CD31 showed 4 to eightfold enrichment in BMV preparations compared to vessel-depleted brain parenchyma; brain-endothelial cell-specific Slc2a1/glucose transporter (Glut1) showed over 60-fold enrichment in BMVs. *Pericyte markers*, platelet-derived growth factor receptor beta (Pdgfrb), desmin (Des), smooth muscle actin (Acta2), and CD13 (aminopeptidase N/Anpep), were also enriched in BMVs (2 to sixfold), whereas *astrocyte markers* glial fibrillary acidic protein (Gfap), protein S100-beta (S100β), electrogenic sodium bicarbonate cotransporter 1 (Slc4a4), and aquaporin-4 (Aqp4) were 2 to fourfold enriched in brain parenchyma compared to BMVs. This data confirms that BMV isolation protocol used in this study yielded endothelial cell- and pericyte-enriched microvessels with significant depletion of astrocytes, although remnants of the astrocytic end-feet could still be detected by in situ immunofluorescence.

### RNA-seq datasets: comparability and validation

Isolated mouse and human BMVs and organs were subjected to RNA-seq analyses; an enrichment of RMT receptors in BMVs was compared to peripheral organs and the whole brain (without vascular depletion).

When RNA-seq data generated in this study for human (total) brain and lung were compared with public RNA-seq data, a strong correlation (Pearson correlation coefficient of 0.96) (Additional file 1: Figure S1A, B) was observed. Comparison of biological replicates (brain total, lung total and brain vessels) analyzed in this study also showed high correlation (Pearson correlation coefficients ranging between 0.94 and 0.97, Additional file 1: Figure 1c–e). These analyses confirmed internal reproducibility and comparability of the RNA-seq data generated in this study with available ‘benchmark’ external datasets.

**Fig. 3:**
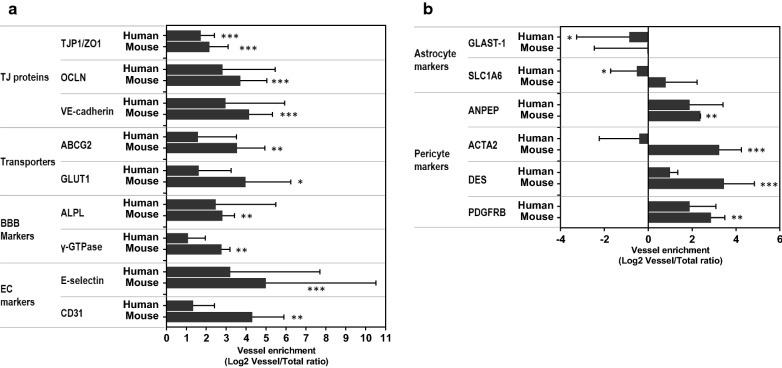
The enrichment of transcript for specific cell types in isolated mouse and human brain microvessels (BMV) compared to whole brain. BMVs and whole brain extracts were isolated as described in Materials and Methods and subjected to RNASeq analyses. **a***Endothelial cell/BBB markers*: tight-junction protein 1 (TJP1)/ZO1, Occludin (OCLN), VE-cadherin, transporters (GLUT1, ABCG2), enzymes (ALP, γ-GTPase), and endothelial cell markers (E-selectin, CD31). **b***Astrocyte*-*specific markers:* GLAST1 and SLC1A2; *Pericyte*–*specific markers:* PDGFRB, DES; ACTA2, ANPEP in human and mouse BMVs and total brain tissue. Each bar represents three biological replicates (Mean ± SEM). The enrichment of a gene transcript in mouse BMV vs. whole brain and in human BMV vs. whole brain was analyzed by two-tailed student *t*-test. Data are shown as Log2 ratios of various genes between levels in vessels and total brain

Further quality control of the dataset was performed by analyzing a relative enrichment of endothelial or BBB-specific gene transcripts in BMVs compared to (total) brain. Gene transcripts encoding *endothelial cell* genes Glut-1, VE-cadherin, E-selectin, CD31, tight-junction protein 1 (TJP1)/ZO1, occludin (OCLN), ABC transporter (ABCG2) and enzymes alkaline phosphatase (ALP) and γ-glutamyl transpeptidase (γ-GTP) were highly enriched in BMVs compared to the total brain in both mouse and human samples (Fig. 3a). Pericyte marker genes similarly were enriched in brain vessels relative to total brain tissues (Fig. 3b); while the transcript abundances of the *astrocyte*-specific glutamate transporters GLAST1 and SLC1A6 were higher in the brain than in the BMV (Fig. 3b).

### The expression of RMT genes in isolated human BMVs, brain and lung

**Fig. 4:**
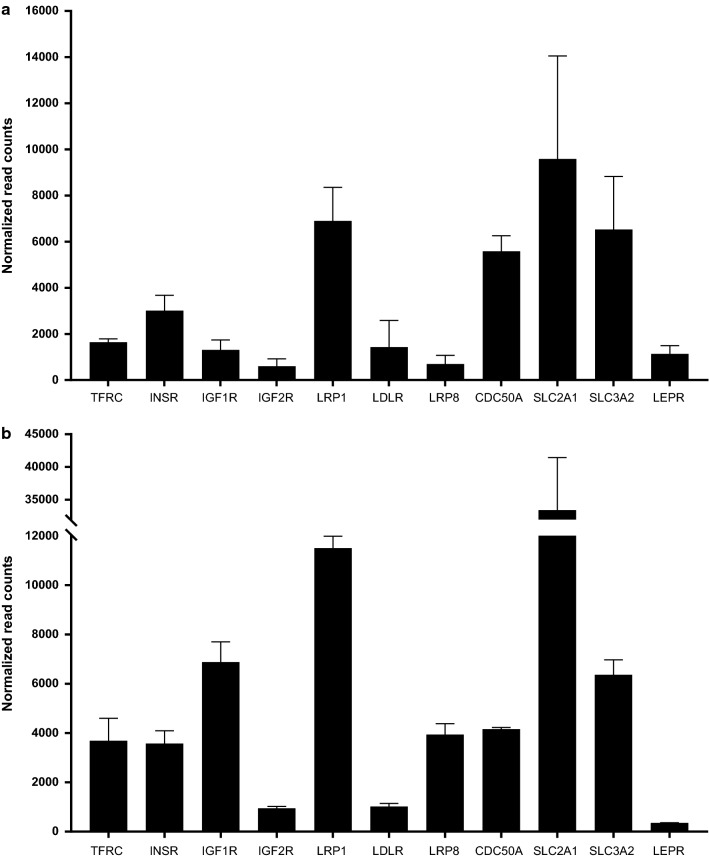
The abundance of RMT receptor and transporter gene transcripts in human (**a**) and mouse (**b**) brain microvessels (BMV). BMVs were isolated as described in Materials and Methods and subjected to RNASeq analyses. Data are shown as normalized read counts (Mean ± SD from three biological replicates). Statistical analyses was performed using one-way ANOVA, followed by Tukey’s multiple comparisons test and p < 0.05 was considered significant. For human BMV (**a**), the level of SLC2A1/GLUT1 is significantly higher than those of IGF2R, LRP8, and LEPR (p < 0.05). The level of SLC3A2/CD98hc is significantly higher than those of LDLR, LRP8, IGFR, IGF2R and LEPR (p < 0.05). The level of LRP1 is significantly higher than those of TFRC, LDLR, IGF1R and LEPR (p < 0.05) and LRP8 and IGF2R (p < 0.01). For mouse BMV (**b**), the level of SLC2A1/GLUT1 is significantly higher than those of TFRC, INSR, IGF1R, IGF2R, LDLR, LRP8, CDC50A, and LEPR (p < 0.0001), and LRP1 (p < 0.001). The level of SLC3A2/CD98hc is significantly higher than those of TFRC and INSR (p < 0.05), LDLR, IGF2R and LEPR (p < 0.0001). The level of LRP1 is significantly higher than those of TFRC, INSR, IGF2R, LDLR, LRP8 and LEPR (p < 0.0001) and CDC50A (p < 0.001). The level of IGF1R is significantly higher than those of TFRC and INSR (p < 0.01), CDC50A (p < 0.05), IGF2R and LEPR (p < 0.0001). The level of TFRC is significantly higher than those of IGF2R (p < 0.05) and LEPR (p < 0.01). The level of LRP8 is significantly higher than those of IGF2R (p < 0.05) and LEPR (p < 0.01). The level of CDC50A is significantly higher than those of IGF2R and LEPR (p < 0.01)

The expression levels of genes encoding RMT receptors in isolated human BMVs, total brain and lung tissues are listed in Table 1. The SLC2A1/GLUT-1 was the most abundant among the genes expressed in human BMVs (Table 1). The rank order of other RMT receptor transcript abundance in human BMVs was LRP1 > SLC3A2/CD98hc > CDC50A/TMEM30A > INSR > TFRC > LDLR > IGF1R > LEPR > LRP8 = IGF2R (Table 1; Fig. 4a). LRP1, SLC3A2/CD98hc and CDC50A were expressed at similar levels, and were significantly higher than all other RMT receptors studied (Fig. 4a).

**Table 1:** Expression levels of genes encoding RMT receptors in isolated human brain microvessels (BMV), brain and lung (RNAseq normalized read counts; average ± SD)

Receptors/proteins	Human BMV	Human brain	Human lung
TFRC	1645.22 ± 249.68	1560.20 ± 520.04	7856.4 ± 6648.12
INSR	3010.84 ± 1158.90*	854.14 ± 81.00	1260.72 ± 106.44
IGF1R	1304.87 ± 746.61	905.46 ± 229.11	800.57 ± 198.75
IGF2R	602.08 ± 550.37	613.27 ± 194.15	4162.89 ± 2237.01*
LRP1	6896.83 ± 2520.15	5797.91 ± 1220.33	12,733.83 ± 4968.54
LDLR	1424.41 ± 2003.73*	294.13 ± 121.19	6952.95 ± 2791.13
LRP8 (ApoER2)	695.27 ± 650.56	734.55 ± 153.52	116.66 ± 69.72
CDC50A/TMEM30A	5578.82 ± 1174.14	7977.31 ± 2267.54	7621.38 ± 3189.82
SLC2A1/GLUT1	9579.89 ± 7741.88^#^	3110.7 ± 1033.78	472.20 ± 217.86
SLC3A2/CD98hc	6527.14 ± 3983.38	4619.39 ± 509.35	5088.29 ± 425.63
LEPR	1131.17 ± 617.91*	1932.75 ± 612.91	3549.49 ± 132.05

Comparison of gene expression (transcript abundance) across isolated human BMVs, brain and lung was performed using One-way ANOVA, followed by Tukey’s multiple comparisons test. Significant difference was indicated by (*). For simplicity, statistical significance was shown only for comparisons of BMV against other tissues (but not among other tissues)

*INSR expression in BMV was significantly (p < 0.05) higher compared to either brain or lung

*IGF2R expression in lung tissue was significantly (p < 0.05) higher compared to either brain vessels or brain

*LDLR expression in BMV was significantly higher (p < 0.001) compared to brain and significantly lower (p < 0.05) compared to lung

^#^SLC2A1/GLUT1: one sample out of 3 showed low transcript level, causing high SD. Therefore, despite overall high abundance, there was no significant difference compared to brain and lung. *LEPR expression in BMV was significantly lower compared to lung (p < 0.001)

INSR showed higher abundance (enrichment) in isolated human BMVs compared to either the brain or the lung (Table 1). SLC3A2/CD98hc, CDC50/TMEM30A and IGF1R were expressed at similar levels in brain vessels, brain and lung; whereas TFRC, LRP1, LDLR, IGF2R and LEPR were comparatively highly enriched in the lung (Table 1).

### The expression of RMT genes in isolated mouse BMVs, lung microvessels, brain, liver and spleen

RNA-seq analyses were performed on isolated mouse BMVs, lung vessels, and whole tissue extracts of the brain, liver and spleen (Table 2; Fig. 4b). The gene showing the highest abundance and selectivity in isolated mouse BMVs was SLC2A1/GLUT1 (Table 2). Among other putative RMT receptors/transporters, the rank order in abundance in mouse BMVs was LRP1 > IGF1R = SLC3A2/CD98hc > CDC50A = LRP8 = TFRC = INSR > LDLR = IGF2R > LEPR (Table 2; Fig. 4b).

**Table 2:** Expression levels of mouse genes encoding RMT receptors in isolated brain microvessels (BMV), whole brain, liver, spleen and lung vessels (RNAseq normalized read counts; average ± SD)

Receptors/proteins	BMV	Brain	Liver	Spleen	Lung vessels
TFRC	3690.57 ± 1577.32*	1366.54 ± 198.69	824.73 ± 70.64	3112.77 ± 1853.22	358.11 ± 79.98
INSR	3577.43 ± 889.85*	956.02 ± 215.79	1219.83 ± 877.37	561.32 ± 212.32	1475.28 ± 84.75
IGF1R	6876.68 ± 1430.93*	1102.77 ± 297.56	38.19 ± 27.33	386.79 ± 70.17	1920.06 ± 367.45
IGF2R	952.45 ± 129.07	705.28 ± 248.38	413.13 ± 188.02	926.11 ± 358.19	1531.46 ± 133.94*
LRP1	11,506.40 ± 833.68*	8489.28 ± 1692.30	4448.88 ± 920.24	3403.74 ± 934.22	6397.87 ± 829.14
LDLR	1015.42 ± 223.35	584.67 ± 170.78	2210.82 ± 1160.86	850.94 ± 230.47	1062.21 ± 116.65
LRP8 (ApoER2)	3937.6 ± 777.50*	1318.88 ± 271.37	15.31 ± 20.31	310.13 ± 107.61	36.57 ± 12.07
CDC50A/TMEM30A	4159.55 ± 119.32*	8495.71 ± 1376.15	13,191.59 ± 2020.86	2457.55 ± 424.88	3534.28 ± 715.38
SLC2A1/GLUT1	33,418.54 ± 13,876.59*^#^	2127.85 ± 246.70	216.14 ± 34.86	1164.86 ± 192.17	478.96 ± 48.42
SLC3A2/CD98hc	6369.22 ± 1046.86*	2665.44 ± 27.16	979.66 ± 147.88	7792.42 ± 1947.03	5596.12 ± 929.74
LEPR/leptin receptor	356.37 ± 14.03*	90.68 ± 1.77	107.97 ± 9.58	667.06 ± 116.16	1917.86 ± 469.42

Comparisons of gene expression (transcript abundance) across isolated mouse BMVs and peripheral tissues was performed using one-way ANOVA, followed by Tukey’s multiple comparisons test. For simplicity, statistical significance was shown only for comparisons of BMVs against other tissues (but not among peripheral tissues)

*TFRC expression in BMV was significantly (p < 0.05) higher compared to lung vessels

*INSR expression in BMV was significantly higher (p < 0.01) compared to brain, liver, lung vessels or spleen

*IGF1R expression in BMV was significantly (p < 0.0001) higher compared to brain, liver, spleen or lung vessels

*IGF2R expression in lung vessels was significantly higher compared to brain tissue (p < 0.01) and liver (p < 0.001)

*LRP1 expression in BMV was significantly (p < 0.01) higher compared to liver, spleen, or lung vessels

*LRP8 expression in BMV was significantly (p < 0.0001) higher compared to brain, liver, spleen or lung vessels

*CDC50A/TMEM30A expression in BMV was significantly (p < 0.01) lower compared to brain or liver

*SLC2A1/GLUT1 expression in brain vessels was significantly (p < 0.001) higher compared to brain tissue, liver, spleen and lung vessels

^#^SLC2A1/GLUT1 expression was significantly (p < 0.001) higher in BMV compared to the expression off all other genes shown in Table 2

*SLC3A2/CD98hc expression in BMV was significantly higher (p < 0.01) compared to brain or liver

*LEPR expression in BMV was significantly (p < 0.0001) lower compared to lung vessels or spleen and significantly (p < 0.001) higher compared to brain or liver

SLC2A1, IGF1R, INSR and LRP8 were distinctly enriched in mouse BMVs compared to the brain, lung vessels and peripheral tissues examined in this study (Table 2). TFRC showed high transcript abundance in both BMVs and the spleen; whereas SLC3A2/CD98hc showed high transcript abundance in the spleen and lung vessels. CDC50A was enriched in the brain, liver and lung vessels (Table 2). IGF2R and LEPR showed relative enrichment in lung microvessels; while LDLR was highly expressed in liver, spleen and lung vessels (Table 2).

### Cellular source of RMT receptor transcripts enriched in mouse and human BMVs

Comparisons of normalized RNA abundance of RMT receptors in human and mouse BMVs (from the current study) and available public datasets obtained from brain endothelial cells, astrocytes and neurons, as well as the whole brain and lung from corresponding species are shown in Additional file 1: Figure S2A, B. Data included in these analyses were obtained by single-cell sequencing of freshly isolated cells from the mouse brain vascular segments [49] and from the fetal and adult human cortex [49–51].

Comparative analyses suggested that the endothelial enrichment in SLC2A1, TfR, INSR, SLC3A2/CD98hc and LRP8 is largely responsible for the high abundance of these genes observed in the isolated mouse BMVs [50, 51]; observed LRP1 expression in mouse BMVs appears to originate from its abundance in pericytes and astrocytes; whereas observed expression levels of IGF1R, CDC50A and SLC2A1/Glut-1 may originate from either one or all three cell types forming the neurovascular unit (NVU). A recent publication by Kalucka et al. [52], which mapped single-cell transcriptome atlas of murine endothelial cells, identified IGF1R, TfR, LRP8 and SLC2A1 as highly enriched in BEC compared to endothelial cells from all other tissues; IGF1R transcript was threefold more abundant than TfR in BEC [52]. Human BMVs analyzed in this study appeared to have lower than expected expression of SLC2A1, TfR, LDLR, LRP8 and IGF1R compared to the endothelial expression of these genes derived from the single cell sequencing of the fetal and adult human cortex [49–51]. Similarly to what was observed with mouse BMVs, high LRP1 expression observed in human BMVs does not appear to originate from endothelial cells.

### Species differences in the expression of RMT receptors in isolated human and mouse BMVs

The expression patterns of RMT receptors in BMVs and the whole brain of human and mouse were compared across species (Table 3). The abundance of receptor transcripts was compared across species using normalized transcripts per million (TPM) counts (Table 3).

**Table 3:** Expression levels of genes encoding RMT receptors in isolated human and mouse brain microvessels (BMV) and whole brain tissues [RNAseq normalized for transcript per million (TPM); Mean ± SD]

Receptors/proteins	Human BMVs	Human brain	Mouse BMV	Mouse brain
TFRC	8.81 ± 4.43	11.25 ± 2.80	70.53 ± 32.21*	20.85 ± 4.41^#^
INSR	5.44 ± 3.70	4.75 ± 0.49	33.09 ± 8.63*	8.08 ± 3.31
IGF1R	2.38 ± 1.65	2.86 ± 0.69	61.06 ± 13.70*	7.36 ± 2.33^#^
IGF2R	3.09 ± 3.42	3.88 ± 2.06	9.88 ± 1.40*	5.86 ± 2.57
LRP1	21.18 ± 16.53	28.80 ± 6.61	179.85 ± 15.66*	69.87 ± 15.36^#^
LDLR	14.04 ± 19.25	5.19 ± 1.55	21.21 ± 5.00	9.67 ± 3.66
LRP8 (ApoER2)	6.09 ± 6.13	5.58 ± 1.75	20.66 ± 5.65	89.11 ± 20.41^#^
CDC50A/TMEM30A	28.62 ± 16.30	68.42 ± 15.01	98.36 ± 1.58*	158.57 ± 13.88^#^
SLC2A1/GLUT1	86.20 ± 88.50	41.64 ± 12.54	1314.53 ± 559.01*	64.62 ± 1.08^#^
SLC3A2/CD98hc	86.68 ± 75.46	89.37 ± 3.74	406.81 ± 66.31*	138.96 ± 14.72^#^
LEPR/leptin receptor	2.25 ± 1.96	1.19 ± 0.32	1.35 ± 1.35	2.10 ± 0.71
GAPDH	1563.77 ± 301.52	3924.81 ± 295.78	1571.31 ± 237.26	2824.45 ± 442.70^&^
S100B	520.09 ± 180.37	1005.79 ± 308.03	547.96 ± 5.82	231.91 ± 33.28^&^
TUBB4A	328.93 ± 126.15	363.04 ± 92.13	357.16 ± 110.37	552.25 ± 194.57

Statistical comparison of gene expression (transcript abundance) between human and mouse BMVs and human and mouse brain was performed using two-tailed student *t*-test. Significant difference between human and mouse BMVs was indicated by (*) and significant difference between human and mouse brain is indicated by (#), *Receptor abundance is significantly higher (TFRC p < 0.01; INSR p < 0.001; IGF1R p < 0.01; IGF2R p < 0.05; LRP1 p < 0.001; LRP8 p < 0.001; CDC50A p < 0.01; SLC3A2 p < 0.01; SLC2A1 p < 0.01) in mouse compared to human BMVs

^#^Receptor expression is significantly higher (TFRC p < 0.05; IGF1R p < 0.05; LRP1 p < 0.05; LRP8 p < 0.005; CDC50A p < 0.002; SLC2A1 p < 0.05; SLC3A2 p < 0.01) in mouse compared to human brain

^&^Genes encoding structural proteins: S100B is significantly (p < 0.001) lower in mouse compared to human brain; GAPDH is significantly lower in mouse compared to human brain (p < 0.05)

The expression levels of SLC2A1, TFRC, LRP1, LRP8, IGF1R, IGF2R, CDC50A/TMEM30A and SLC3A2/CD98hc were all significantly higher in mouse BMVs compared to human BMVs (Table 3). The expression levels of LRP8, LDLR and LEPR were not significantly different in BMVs between two species (Table 3). LRP1, LRP8 and CDC50A/TMEM30A were more enriched in the mouse compared to human brain (Table 3).

The structural genes (GAPDH and S100B) were similarly expressed in mouse and human BMVs, and TUBB4A was expressed at similar levels in human and mouse BMVS and brains (Table 3).

### IGF1R, LRP1 and TfR protein expression in human and mouse BMVs

Some of the above findings were validated at the protein expression level using the same BMV preparations that were analyzed by RNA-seq. Due to limited amount of BMV samples and based on the availability of species cross-reactive antibodies, three receptors were chosen against which antibodies/ligand were developed and tested as BBB-carriers in clinical (TfR, LRP-1) or advanced preclinical (IGF1R) studies. All three RMT receptors showed higher transcript abundance in mouse BMVs by RNA-seq. The protein expression of these RMT receptors was analyzed by Western blot (Wes™) and immunodetection.

**Fig. 5:**
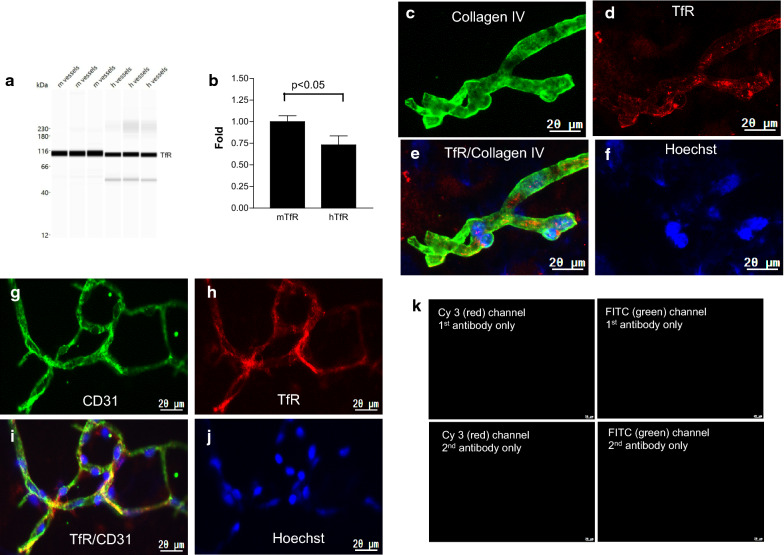
TfR protein expression in isolated brain microvessels (BMVs). **a** Wes™ band density of TfR expression in human (h) and mouse (m) BMVs. **b** Quantification of TfR expression by Wes™ TfR in each lane is normalized to β-actin in the same lane; the fold-change of human TfR is relative to mouse TfR (set as one). Bars are mean ± SD of three biological replicates. The level of mTfR is significantly higher than hTfR (p < 0.05; unpaired two-tailed *t*-test). **c–f** Collagen IV (green) and TfR (red) immunofluorescence in human BMV. **g–j** CD31 (green) and TfR (red) immunofluorescence in mouse BMV. Nuclei are stained with Hoechst (blue) (**f** and **j**). **k** Negative controls for G**–**J. The scale bar is 20 µm

TfR protein expression was higher in mouse BMVs compared to human BMVs by Wes™ analyses (Fig. 5a, b; *t*-test p < 0.05) (normalized to β-actin). TfR was detected by immunofluorescence in both mouse (Fig. 5c–e) and human (Fig. 5f–h) BMVs.

**Fig. 6:**
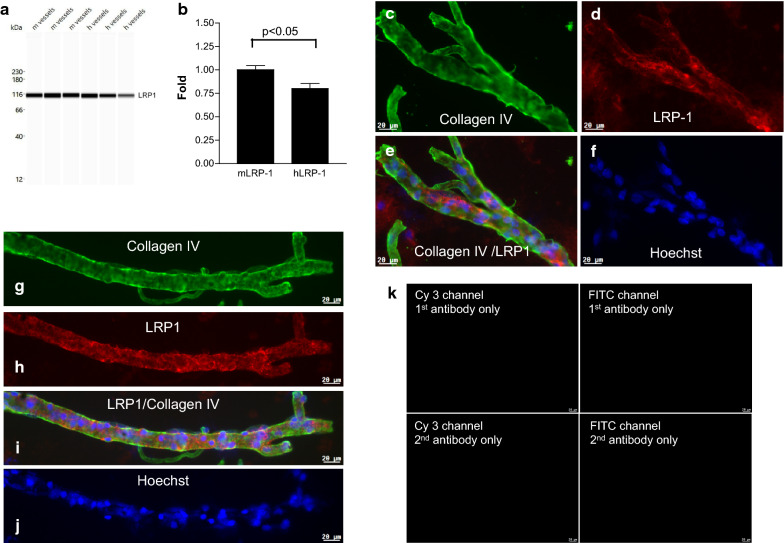
LRP-1 protein expression in isolated brain micrvessels (BMVs). **a** Wes™ band density of LRP-1 expression in human (h) and mouse (m) BMVs. **b** Quantification of LRP1 expression by Wes™. LRP-1 in each lane is normalized to β-actin in the same lane; the fold-change of human LRP-1 is relative to mouse LRP-1 (set as one). Bars are mean ± SD of three biological replicates. The level of mLRP-1 is significantly higher than hLRP-1 (p < 0.05; unpaired two-tailed *t*-test). **c–f** Collagen IV (green) and LRP-1 (red) immunofluorescence in human BMV. **g–i** Collagen IV (green) and LRP-1 (red) immunofluorescence in mouse BMV. **j** Nuclei are stained with Hoechst (blue). **k** Negative controls for G-J. The scale bar is 20 µm

The protein levels of LRP1 were also higher in mouse BMVs compared to human BMVs by Wes™ analyses (Fig. 6a, b; *t*-test p < 0.05) (normalized to β-actin); strong immunofluorescence was detected in BMVs from both species around cell nuclei and borders and, occasionally, overlapping with collagen IV immunofluorescence (Fig. 6 d, e, h, i).

**Fig. 7:**
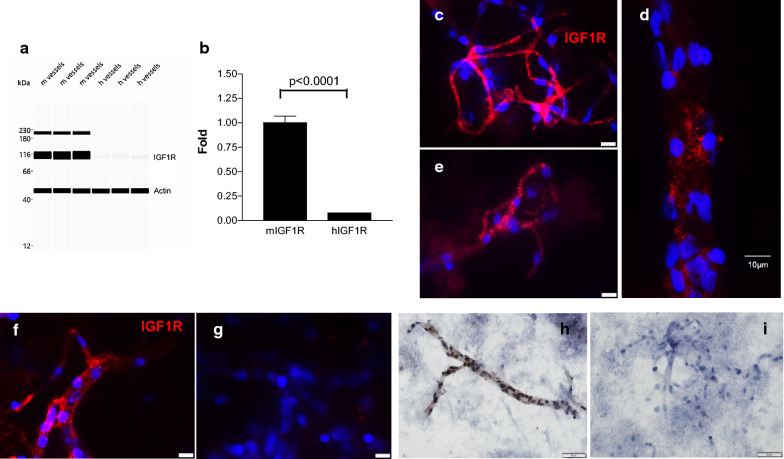
IGF1R protein expression in brain microvessels (BMVs). **a** Wes™ band density of IGF1R expression in human (h) and mouse (m) BMVs. **b** Quantification of IGF1R expression by Wes™. IGF1R in each lane is normalized to β-actin in the same lane; the fold-change of human IGF1R is relative to mouse IGF1R (set as one). Bars are mean ± SD of three biological replicates. The level of mIGF1R is significantly than hIGF1R (p < 0.0001; unpaired two-tailed *t*-test). **c–e** IGF1R (red) immunofluorescence in mouse BMV. **f–g** IGF1R immunofluorescence in human BMVs. Nuclei were stained with Hoechst (blue). The scale bar is 20 µm (unless indicated differently in the micrographs). **h** IGF1R expression (brown) in human BMVs detected by immunohistochemistry. **i** Negative control (no primary antibody) for Panel H. Scale bar for H and I is 10 µm

Western blot (Wes™) analysis demonstrated significantly lower expression of IGF1R in human compared to mouse BMVs (Fig. 7a, b; *t*-test p < 0.0001) (normalized to β-actin). Immunofluorescence (Fig. 7c–g) and immunohistochemistry (Fig. 7h–i) analyses demonstrated a strong expression of IGF1R in both mouse (Fig. 7c, d) and human (Fig. 7e–i) BMVs, often observed as punctate, vesicular immunoreactivity in vessel walls. Immunostaining studies could not detect differences in IGF1R expression between mouse and human BMVs.

## Discussion

Antibodies or peptide ligands against brain endothelial cell receptors that undergo RMT have been developed for brain delivery of therapeutic cargos. This strategy exploits a natural transport function of these receptors, which supply the brain with essential proteins (such as transferrin, insulin and insulin-like growth factors), peptides (such as leptin), essential amino acids, glucose or lipids. However, most of these receptors also play important functions and are expressed broadly or selectively in peripheral organs and tissues. In addition, their expression levels and distribution may vary in different species or pathologies, confounding translational development of BBB delivery carriers. The expression levels of genes encoding RMT receptors in different tissues and species have not been systemically investigated.

The selection of RMT receptors or other transporters for the development of BBB shuttles (carriers) should be guided by several criteria, including a) the selectivity of the expression in BEC compared to peripheral tissues and organs, to assure good systemic pharmacokinetics and to mitigate potential toxicity; b) low expression in brain parenchymal cells to avoid off-target distribution of the cargo, especially when cargo is an antibody against parenchymal target; c) abundance of the receptor in BEC to assure a sufficient transport capacity for a given application; c) similar patterns of expression in both BEC and other organs in experimental pre-clinical species and in humans to ensure translation of experimental findings to clinical application; d) the ability of ligands (natural or engineered) to the receptor to internalize into and to transcytoses/release on the abluminal side of the brain endothelial cells.

To facilitate translational studies of BBB RMT carriers (shuttles) developed against various RMT receptors described in the literature (Additional file 1: Table S1), we have compared their abundance and enrichment in isolated mouse or human brain microvessels (BMVs) to those in either the whole brain or peripheral tissues. The study revealed a significant species differences in selected RMT receptor abundance in different organs, as well as in BMVs.

The case study of TfR antibodies illustrates important translational hurdles in the pre-clinical to clinical development path of BBB carrier antibodies. TfR antibodies with various characteristics have been developed for delivery of biotherapeutic cargos across the BBB, notably TfR/BACE1 bispecific antibody [7] and an anti-Aβ antibody fused to a single TfR-binding Fab fragment [37]. TfR is highly expressed in reticulocytes causing on-target toxicity of effector-function competent antibodies [38]; high-affinity TfR antibodies also exhibit poor pharmacokinetics due to peripheral target-mediated clearance [42, 53]. Due to lack of species cross-reactivity of TfR antibodies, the development of transgenic mouse expressing human TfR extracellular domain [7, 12, 13, 37], use of surrogate antibody or of transgenic mouse expressing human TfR [14, 15, 37] were necessary in pre-clinical studies. The translational PK-PD models developed based on these studies [54] did not take into account differences in TfR abundance between mouse and human. Comparative analyses of the TFRC expression performed in this study demonstrated a significantly higher abundance of TFRC in the mouse compared to human BMVs. In both human and mouse BMVs, TfR abundance was lower than those of SLC3A2/CD98hc and LRP1. Clinical trials with TfR antibodies as BBB carriers have been initiated by Roche (to deliver Aβ-antibody for treatment of Alzheimer’s disease) and by JCR Pharma (to deliver iduronate-2-sulfatase enzyme for treatment of Mucopolysaccharidosis II) [42]. These studies will provide further understanding of how differential TfR abundance in the brain vasculature, the brain and peripheral tissues between pre-clinical mouse species and humans may influence pharmacokinetics, toxicity and brain exposure of TfR antibody-containing biologics tested in the clinic.

Our study further found that: 1) INSR, IGF1R, LRP1 and LRP8 were enriched in the mouse BMVs compared to peripheral tissues; and INSR was enriched in human BMVs compared to total brain and lung; 2) SLC2A1/GLUT1, LRP-1, SLC3A2/CD98hc and CDC50A were the most abundant among receptor/transporters studied in human BMVs; whereas SLC2A1/GLUT1, LRP-1, SLC3A2/CD98hc and IGF1R were the most abundant in mouse BMVs; 3) mouse BMVs have significantly higher abundance of TfR, INSR, IGF1R, LRP-1, LRP8 and CDC50A compared to human BMVs.

The caveat of the current study is that RMT gene transcripts were analyzed in isolated BMVs which contain more than one cell type. A strong endothelial enrichment in our BMV preparations has been confirmed by RNA-seq, targeted proteomics and immunofluorescence analyses; however, the presence of contaminating pericytes and astrocytic end-feet still confound the interpretation of the results. Therefore it is important to interpret results from this study in conjunction with the data obtained by other published studies that analyzed transcript profiles in brain cells or vascular segments using single-cell sequencing [20, 49, 52]. Human BMVs from this study showed comparatively lower expression of TfR, IGF1R, LRP8, LDLR and LEPR from that found in BEC obtained by the single-cell sequencing of human brain [20]. While transcript dilution as a result of the presence of multiple cell types, notably pericytes, in microvessel preparations compared to the purified cells, the other factors such as age of the donors, could also contribute to this observation. In contrast, RMT receptor expression in mouse BMVs was comparatively similar to that observed in BEC by the single-cell sequencing of mouse brain vessels [19], with the exception of the higher expression of LRP1 in isolated BMVs. Single-cell sequencing studies of human and mouse brain and brain vessels [19, 21] both found LRP-1 expression to be very low in BEC, in contrast to high expression in brain vascular smooth muscle cells, pericytes and fibroblast-like cells. A study in cultured bovine NVU cells showed eightfold higher expression of LRP-1 in pericytes compared to brain endothelial cells [55]. This suggests that, despite being among the most abundant transcripts in isolated BMVs, LRP-1 expression originates from non-endothelial cells. This also brings into question its postulated role as BEC RMT receptor useful for therapeutic delivery across the BBB; it should be noted that antibodies raised against LRP1 failed to show facilitated BBB crossing [34].

Using proteomic survey, Zuchero et al. [34] found a very high abundance of basigin (BSG), followed by TfR and SLC3A2/CD98hc, whereas LRP1 was expressed at very low levels in freshly isolated endothelial cells from the mouse brain. RNA-seq data of mouse BMVs in this study ranked the abundance of SLC3A2/CD98hc higher than that of TfR, and similar to that of IGF1R. SLC3A2/CD98hc was also strongly expressed in the mouse spleen, consistent with the reported high expression levels in lymphocytes [56]. In contrast, SLC3A2/CD98hc expression was low in both human and mouse whole brain tissues.

Among the putative RMT receptors analyzed, SLC2A1 and IGF1R showed the highest selectivity of expression in mouse BMVs compared to all peripheral tissues, as well as the whole brain. IGF1R transcript levels were twofold more abundant than the TfR in mouse BMVs and were similar to that of the TfR in human BMVs. A recent single-cell transcriptomics study of endothelial cells in various mouse vascular beds [52], also found IGF1R highly enriched in BEC and threefold more abundant than TfR. In contrast, IGF2R exhibited low abundance in BMVs and high levels in lung tissues. This is consistent with the known developmental down-regulation of IGF2R in adult BBB, also accompanied by the lost ability of the receptor to facilitate transport of mannose 6-phosphate enriched lysosomal storage enzymes into the brain [57].

The additional caveat of transcriptomics studies is that the protein expression/function does not always correlate with the transcript abundance in given tissues. For example, a quantitative proteomics study of transporters and receptor in isolated human BMVs observed higher expression of TfR (2.34 ± 0.76 fmol/µg protein) compared to either LRP-1 (1.51 ± 0.26 fmol/µg protein) or INSR (1.09 ± 0.21 fmol/µg protein) [58], whereas in this study the LRP-1 transcript abundance in human BMVs was the highest among studied putative RMT receptors; albeit likely due to its enrichment in pericytes. Our within-study comparisons between the transcript abundance and the protein expression by Wes analysis for TfR, LRP-1 and IGF1R showed similar trends of lower expression of IGF1R and TfR in human compared to mouse BMVs; whereas no difference was found for LRP1. A species cross-reactive antibodies used in these studies make true quantification of expression levels difficult; for example immunodetection studies with the same antibodies did not detect appreciable expression differences between human and mouse BMVs, likely because antigen retrieval and antibody reactivity to antigenic epitopes in two methods are very different.

## Conclusion

This study provides a molecular transcriptomics map of key RMT receptors in mouse and human brain microvessels, brain and peripheral tissues, important to translational studies of biodistribution, efficacy and safety of antibodies developed against these receptors.

RMT receptor genes in brain microvessels, brain parenchyma and peripheral organs from human and mouse were analyzed by RNA-seq and validated by Western blot and in situ immunodetection. SLC2A1, IGF1R, INSR and LRP8 were highly enriched in mouse BMVs compared to peripheral tissues; additionally SLC2A1 and IGF1R showed high abundance in mouse BMVs. Antibodies against IGF1R have been shown to internalize into brain endothelial cells; while antibodies against both these targets (SLC2A1 and IGF1R) showed enhanced total brain levels in mice compared to control antibodies (although in the absence of vessel depletion for SLC2A1 antibodies). Among evaluated receptors, IGF1R and SLC2A1 appear to satisfy most of the criteria for promising targets to develop trans-BBB delivery ligands; however, it should be understood that SLC2A1 is a carrier-mediated transporter unlikely to undergo a typical RMT. In addition to desired characteristics of the target receptor, development and optimization of an optimized ligand/antibody against these targets is essential for achieving success in improved delivery of biotherapeutics across the BBB.

Cross-species differences in the expression of RMT in BMVs, brain and peripheral tissues is an important consideration in translational studies to advance BBB carriers developed against these receptors into clinical trials. This study suggests that the mouse BBB expresses many of targeted RMT receptors at higher levels than the human BBB. Since the mouse is the most widely used pre-clinical model for discovery and evaluation of investigational compounds, including brain delivery ‘shuttles’, translational studies and PK-PD modeling to estimate dosing in humans need to account for species differences in RMT receptor abundance in BEC and different organs.

## Supplementary information

**Additional file 1.** Additional figures and tables.

## Data Availability

The RNA-seq data is presented in Tables 1, 2 and 3, and the dataset used and/or analyzed in this study is available from the corresponding author on reasonable request.

## Abbreviations

BBB: Blood–brain barrier
BEC: Brain endothelial cells
BMV: Brain microvessels
GFAP: Glial fibrillary acidic protein
GLUT-1/SLC2A1: Glucose transporter-1
γ-GTP: γ-Glutamyl transpeptidase
INSR: Insulin receptor
IGF1R: Insulin-like growth factor-1 receptor
IGF2R: Insulin-like growth factor-2 receptor
LDLR: Low-density lipoprotein receptor
LRP-1: LDLR-related protein 1
LRP8: LDLR-related protein 8
LEPR: Leptin receptor
RMT: Receptor-mediated transcytosis
CMT: Carrier-mediated transcytosis
TMEM30A/CDC50A: Transmembrane protein 30A
TFRC: Transferrin receptor (gene)
TfR: Transferrin receptor (protein)
