# Supplementary material for: Differential expression of receptors mediating receptor-mediated transcytosis (RMT) in brain microvessels, brain parenchyma and peripheral tissues of the mouse and the human
Source: Fluids Barriers CNS. 2020 Jul 22;17:47. doi: 10.1186/s12987-020-00209-0 (PMC7376922; doi:10.1186/s12987-020-00209-0)

Table 1. Receptors and proteins mediating receptor-mediated transcytosis (RMT) or carrier-mediated transcytosis (CMT) at the blood-brain barrier.

RMT receptor (class)	Target biology	BBB carriers
**Iron transporter**		
Transferrin receptor (TFRC)	Transferrin receptor (TfR) is a carrier protein for transferrin. It imports iron into the cell (via a receptor-mediated endocytosis) and is regulated in response to intracellular iron concentration. TfR1/CD71 encoded by TFRC is a transmembrane glycoprotein composed of two disulfide-linked monomers joined by two disulfide bonds. Each monomer binds one holo-transferrin molecule creating an iron-Tf-TfR complex which enters the cell by endocytosis and crosses the BBB by transcytosis.	Various rat-, mouse, and human-specific antibodies against TfR have been engineered as BBB carriers for therapeutic cargos, including bi-specific antibodies [1,2]. An anti-human TfR antibodies have entered Phase 1 clinical trials for brain delivery of Aβ-binding antibody (Roche) and iduronate-2-sulfatase for patients with Hunter syndrome (JCR Pharmaceuticals Co., Ltd).
**Insulin transporters**		
Insulin receptor (INSR)	INSR is receptor tyrosine kinase which mediates the pleiotropic actions of insulin. Binding of insulin leads to phosphorylation of several intracellular substrates. Phosphorylation of IRSs proteins leads to the activation of two main signaling pathways: the PI3K-AKT/PKB pathway, which is responsible for most of the metabolic actions of insulin, and the Ras-MAPK pathway involved in control of cell growth and differentiation. In addition to binding insulin, the insulin receptor can bind insulin-like growth factors (IGF1 and IGF2). Insulin receptor is expressed highly in the liver and intestine, and moderately in pancreas and choroid plexus (mice) [3].	A humanized chimeric antibody against insulin receptor is developed by Armagen Inc [4-6] and is in clinical trials as brain delivery ‘carrier’ for lysosomal enzymes in patients with Hunter and Hurler syndromes [5]. Insulin receptor antibody-enzyme fusions show fast systemic pharmacokinetics and moderate agonistic action on insulin receptor [5].

Insulin Growth Factor 1 Receptor (IGF1R)	IGF1R is receptor tyrosine kinase which mediates actions of insulin-like growth factor 1 (IGF1). IGF1R binds IGF1 with high affinity and IGF2 and insulin (INS) with low affinity. Ligand binding activates the receptor kinase, leading to receptor autophosphorylation, and tyrosine phosphorylation of multiple substrates, and subsequent activation of two main signalling pathways: the PI3K-AKT/PKB pathway and the Ras-MAPK pathway; receptor activation is involved in cell growth and survival control.<br>The transport of circulatory IGF1 across the BBB *in vivo* is modulated by local neuronal activity [7].	Humanized camelid single-domain antibodies against IGF1R, which do not interfere with IGF1 binding to the receptor have been shown to cross the BBB via receptor-mediated transport [8,9]. Selected IGF1R V_HHs with species cross-reactivity demonstrated a saturable, energy-dependent transport across the human BBB model *in vitro* and highly enhanced brain and CSF exposure in rats [10].
Insulin-like growth factor 2 receptor (IGF2R)/cation-independent mannose-6-phosphate receptor	The cation-independent mannose-6-phosphate/insulin-like growth factor-II receptor (IGF2R) is a membrane-bound glycoprotein consisting of 15 homologous extracellular repeat domains. The major function of this receptor is trafficking of mannose-6 phosphate (M6P)-containing lysosomal enzymes from the trans-Golgi network to the endosomes and their subsequent transfer to lysosomes. The IGF2R also plays a major role in binding and regulating the circulating and tissue levels of IGF2.<br><br>The IGF2R gene is developmentally regulated. Evidence suggest that IGF2R is expressed at the BBB in early postnatal life, but is downregulated in adult BBB.	IGF2R is tested as potential 'shuttle' for M6P enriched lysosomal enzymes for treating lysosomal storage diseases. Some cross-BBB transport was demonstrated in mice in early postnatal age, but was completely lost in adult mice [11]. Re-induction of IGF2R expression in adult brain vessels was suggested as a strategy to improve enzyme delivery.
**Lipid transporters**		
Low-density lipoprotein receptor (LDLR)	LDL-R is a mosaic protein of 839 amino acids that mediates the endocytosis of cholesterol-rich LDL. It recognizes apoprotein B100, which is embedded in	Peptide ligands binding to LDLR were identified using phage-display screening approach. Two lead peptides

	the outer phospholipid layer of LDL particles, as well as apoE protein in chylomicron remnants and VLDL remnants (IDL). In humans, the LDL receptor protein is encoded by the *LDLR* gene and belongs to the Low density lipoprotein receptor gene family [12].	(VH434 and VH4127) have been shown to mediate LDLR-dependent BBB transcytosis [13].
Low-density lipoprotein receptor-related protein 1 (LRP1)	LRP1 is a plasma membrane protein involved in receptor-mediated endocytosis. In humans, the LRP1 protein is encoded by the *LRP1* gene. LRP1 is also a signalling protein involved in lipoprotein metabolism and cell motility, as well as in neurodegenerative diseases, atherosclerosis, and cancer. LRP-1 binds numerous ligands, including alpha2-macroglobulin, amyloid β, APOE and others.	LRP-1 has been implicated in BBB receptor-mediated transport of melanotransferrin [14] and peptide ligands (Aprotinin, Angiopep2) [15]. Angiochem Inc, a developer of angiopep2-paclitaxel conjugate has initiated Phase III clinical trials for treatment of brain tumors.<br><br>Antibodies raised against LRP-1 [16] failed to show enhanced brain uptake compared to control antibodies.
Low-density lipoprotein receptor-related protein 8 (LRP8/ApoER2)	LRP8 is a cell surface receptor belonging to the LDL receptor family which participates in endocytosis and signal transduction. It co-localises with the vascular endothelial cell marker CD31/Pecam1 in mouse brain [17]. Through interactions with one of its ligands, reelin, LRP8 plays an important role in embryonic neuronal migration and postnatal long-term potentiation. Decreased expression of LRP8 is associated with certain neurological diseases.	Fluorescence-labeled anti-LRP8 antibody was shown to transport across the BBB into mouse brain [18].
Transmembrane protein 30A (TMEM30A)/CDC 50A/P4 flippase	TMEM30A is a β subunit (transport to plasma membrane) of a catalytic P4-ATPase flippase. P4-ATPase flippase complex catalyzes hydrolysis of ATP coupled to the transport of aminophospholipids from the outer to the inner leaflet of various membranes and ensures the maintenance of asymmetric distribution of phospholipids. Phospholipid translocation has been implicated in	A putative receptor of the BBB-crossing single-domain antibody FC5 [20,21], isolated from llama VhH libraries by function-first panning [22]. FC5 and its humanized variants have been fused with various centrally acting payloads, including neuropeptides and full monoclonal antibodies [23-25].

	vesicle formation and in uptake of lipid signalling molecules [19]	
**Solute carriers**		
SLC2A1/GLUT1	SLC2A1, also known as glucose transporter 1 (GLUT1), is a uniporter membrane protein encoded by the *SLC2A1* gene. SLC2A1 facilitates the transport of glucose across the plasma membrane and is among the most abundant genes/proteins expressed at the blood-brain barrier.	An antibody raised against SLC2A1 by Genentech [16] showed a three-fold higher levels in the total brain extracts 1 h after iv administration, compared to control antibody.<br>Glucosylated nanocarrier (micells) was developed to target GLUT1 for delivering siRNA across the mouse BBB [26].
SLC3A2/CD98hc	SLC3A2 comprises the heavy subunit of the large neutral amino acid transporter (CD98hc). SLC3A2 is a transmembrane protein and exists as the heavy chain of a heterodimer, covalently bound through di-sulfide bonds to one of several possible light chains. It associates with integrins and mediates integrin-dependent signaling related to normal cell growth and tumorigenesis.	Bispecific antibodies targeting CD98hc and BACE1 were developed and shown to have brain exposure and pharmacodynamic effects on Aβ levels in transgenic mice similar to that achieved by TfR-BACE1 bispecific antibodies [27].
**Neuroactive peptide receptors**		
Leptin receptor (LEPR)	Leptin receptor is a single-transmembrane domain Type I cytokine receptor that functions as a receptor for the fat cell-specific hormone leptin. The leptin regulates adipose-tissue mass through hypothalamic effects on hunger and energy use.	Leptin-derived peptides bind to leptin receptor and the complex is transported across the BBB [25]. A leptin-derived peptide was used to modify pegylated nanoparticles carrying DNA plasmid, resulting in high efficiency of gene delivery into the brain [28]

1. Pardridge WM, et al. Selective transport of an anti-transferrin receptor antibody through the blood-brain barrier *in vivo*. J. Pharma & Exp. Therapeutics 1991; 259: 66-70.
2. Hultqvist G, Syvänen S, Fang XT, Lannfelt L, Sehlin D. Bivalent Brain Shuttle Increases Antibody Uptake by Monovalent Binding to the Transferrin Receptor. Theranostics. 2017; 7(2): 308-18.

3. Watanabe M, Hayasaki H, Tamayama T, Shimada M. Histologic distribution of insulin and glucagon receptors. Braz J Med Biol Res. 1998; 31(2): 243-56.
4. Boado RJ, Pardridge WM. Brain and organ uptake in the rhesus monkey in vivo of recombinant iduronidase compared to an insulin receptor antibody-iduronidase fusion protein. Mol Pharm. 2017; 14(4):1271-77.
5. Boado, R. Platform technology for treatment of brain disorders with blood-brain barrier penetrating IgG-fusion proteins: preclinical and clinical updates. The CHI's 5th Annual Blood-Brain Barrier Meeting, World Pharma Week, June 19 – 20, 2019 (Boston).
6. Pardridge WM, Boado RJ, Giugliani R, Schmidt M. Plasma Pharmacokinetics of Valanafusp Alpha, a Human Insulin Receptor Antibody-Iduronidase Fusion Protein, in Patients with Mucopolysaccharidosis Type I. BioDrugs. 2018; 32(2):169-76.
7. Nishijima T, Piriz J, Duflot S, Fernandez AM, Gaitan G, Gomez-Pinedo U, Verdugo JM, Leroy F, Soya H, Nuñez A, Torres-Aleman I. Neuronal activity drives localized blood-brain-barrier transport of serum insulin-like growth factor-I into the CNS. Neuron. 2010; 67(5): 834-46.
8. Stanimirovic DB, Sandhu JK, Costain WJ. Emerging technologies for delivery of biotherapeutics and gene therapy across the blood-brain barrier. BioDrugs. 2018; 32(6): 547-59.
9. Stanimirovic D, Kemmenrich K, Haqqani AS, Sulea T, Arbabi-Ghahroudi M, Massie B, Gilbert R. Insulin-like growth factor 1 receptor -specific antibodies and uses thereof. WO2015131256A1. 2015.
10. Ribecco-Lutkiewicz M, Sodja C, Haukenfrers J, Haqqani AS, Ly D, Zachar P, Baumann E, Ball M, Huang J, Rukhlova M, Martina M, Liu Q, Stanimirovic D, Jezierski A, Bani-Yaghoub M. A novel human induced pluripotent stem cell blood-brain barrier model: Applicability to study antibody-triggered receptor-mediated transcytosis. Sci Rep. 2018; 8(1): 1873.
11. Urayama A, Grubb JH, Sly WS, Banks WA. Developmentally regulated mannose 6-phosphate receptor-mediated transport of a lysosomal enzyme across the blood-brain barrier. Proc Natl Acad Sci U S A. 2004; 101(34):12658-12663. doi:10.1073/pnas.0405042101
12. Masliah E, Spencer B. Applications of ApoB LDLR-Binding Domain Approach for the Development of CNS-Penetrating Peptides for Alzheimer's Disease. Methods Mol Biol. 2015; 1324:331-37.
13. Jacquot, G. et al. Optimization and in vivo validation of peptide vectors targeting the LDL receptor. Mol. Pharm. 2016; 13: 4094–4105.
14. Demeule M, Poirier J, Jodoin J, et al. High transcytosis of melanotransferrin (P97) across the blood-brain barrier. J Neurochem. 2002; 83: 924-33.
15. Demeule M, Régina A, Ché C, Poirier J, Nguyen T, Gabathuler R, Castaigne JP, Béliveau R. Identification and design of peptides as a new drug delivery system for the brain. J Pharmacol Exp Ther. 2008; 324(3): 1064-72.
16. Zuchero Y, Chen X, Bien-Ly N, Bumbaca D, Tong RK, Gao X, Zhang S, et al. Discovery of Novel Blood-Brain Barrier Targets to Enhance Brain Uptake of Therapeutic Antibodies. Neuron. 2016; 89: 70-82.

17. Daneman R, Zhou L, Agalliu D, Cahoy JD, Kaushal A, Barres BA. The mouse blood-brain barrier transcriptome: a new resource for understanding the development and function of brain endothelial cells. PLoS One 2010; 5: e13741.
18. Watts RJ, Yu J, Dennis M, Freskgard P-O, Tam S. Delivery system for diagnostic and therapeutic agents. Patent publication number CA 2781733 A1, 2011.
19. Coleman JA, Molday RS. Critical role of the beta-subunit CDC50A in the stable expression, assembly, subcellular localization, and lipid transport activity of the P4-ATPase ATP8A2. J Biol Chem. 2011; 286: 17205–16.
20. van der Mark VA, de Waart DR, Ho-Mok KS, et al. The lipid flippase heterodimer ATP8B1-CDC50A is essential for surface expression of the apical sodium-dependent bile acid transporter (SLC10A2/ASBT) in intestinal Caco-2 cells. Biochim Biophys Acta. 2014;1842 (12 Pt A):2378–86.
21. Abulrob A, Brunette E, Slinn J, Baumann E, Stanimirovic D. *In vivo* time domain optical imaging of renal ischemia-reperfusion injury: discrimination based on fluorescence lifetime. Mol Imaging. 2007; 6(5): 304-14.
22. Muruganandam A, Tanha J, Narang S, Stanimirovic D. Selection of phage-displayed llama single-domain antibodies that transmigrate across human blood-brain barrier endothelium. FASEB J. 2002; 16: 240-42.
23. Haqqani, A. et al. Multiplexed evaluation of serum and CSF pharmacokinetics of brain-targeting single-domain antibodies using a NanoLC-SRM-ILIS method. Mol Pharm. 2013; 10: 1542-56.
24. Farrington GK, Caram-Salas N, Haqqani AS, et al. A novel platform for engineering blood-brain barrier-crossing bispecific biologics. FASEB J. 2014; 28: 4764-78.
25. Barrett GL, Trieu J, Naim T. The identification of leptin-derived peptides that are taken up by the brain. Regul Pept. 2009; 155(1-3):55-61.
26. Anraku, Y et al. Glycaemic control boosts glucosylated nanocarrier crossing the BBB into the brain. Nat Commun. 8:1001, 2017.
27. Zuchero Y, Chen X, Bien-Ly N, Bumbaca D, Tong RK, Gao X, Zhang S, et al. Discovery of Novel Blood-Brain Barrier Targets to Enhance Brain Uptake of Therapeutic Antibodies. Neuron. 2016; 89: 70-82.
28. Liu Y, Li J-F, Shao K, Huang R-Q, Ye L-Y, Lou J-N, Jiang C. A leptin derived 30-amino-acid peptide modified pegylated poly-l-lysine dendrigraft for brain targeted gene delivery. Biomaterials 2010; 31 (19): 5246-57.

Supplementary Figure Legends

Supplementary Figure 1. RNA-seq data quality and correlation within and between datasets. Comparative analyses were conducted between RNA-seq data generated in this study (NRC data) and the data from the public domain for human total brain (A) and lung (B) as well as between replicates in human total brain (C), brain vessels (D) and lung (E). Each plot represents the expression of all protein-coding genes; the red line correspond to a linear regression between log2 normalized read counts in each comparison.

Supplementary Figure 2: A) Heat maps showing normalized RNA abundance of the indicated genes in human BMVs compared to public datasets. Legends: BMVs, human brain vessels isolated in this study; Brain endothelial cells (EC), Astrocytes and Neurons are from single cell RNAseq analysis from Barres and co-workers [1, 2]; Brain from whole brain; Lung from whole lung tissues. **B)** Heat maps showing normalized RNA abundance of the indicated genes in mouse BMVs compared to public datasets. Legends: BMVs, mouse brain vessels isolated in this study; Brain EC, Pericytes, Astrocytes and Lung EC are from single cell RNAseq from Betsholtz and co-workers [3, 4].

References:

1. Darmanis S, Sloan SA, Zhang Y, Enge M, Caneda C, Shuer LM, Hayden Gephart MG, Barres BA, Quake SR. A survey of human brain transcriptome diversity at the single cell level. Proc Natl Acad Sci U S A. 2015; 112(23):7285-90.
2. Daneman R, Zhou L, Agalliu D, Cahoy JD, Kaushal A, Barres BA. The mouse blood-brain barrier transcriptome: a new resource for understanding the development and function of brain endothelial cells. PLoS One 2010; 5: e13741.
3. Vanlandewijck M, He L, Mäe MA, Andrae J, Ando K, Del Gaudio F, Nahar K, Lebouvier T, Laviña B, Gouveia L, Sun Y, Raschperger E, Räsänen M, Zarb Y, Mochizuki N, Keller A, Lendahl U, Betsholtz C. Author Correction: A molecular atlas of cell types and zonation in the brain vasculature. Nature. 2018 560(7716):E3. (2018; 554**:** 475-480)
4. He L, Vanlandewijck M, Mäe MA, Andrae J, Ando K, Del Gaudio F, Nahar K, Lebouvier T, Laviña B, Gouveia L, Sun Y, Raschperger E, Segerstolpe Å, Liu J, Gustafsson S, Räsänen M, Zarb Y, Mochizuki N, Keller A, Lendahl U, Betsholtz C. Single-cell RNA sequencing of mouse brain and lung vascular and vessel-associated cell types. Sci Data. 2018 5:180160.

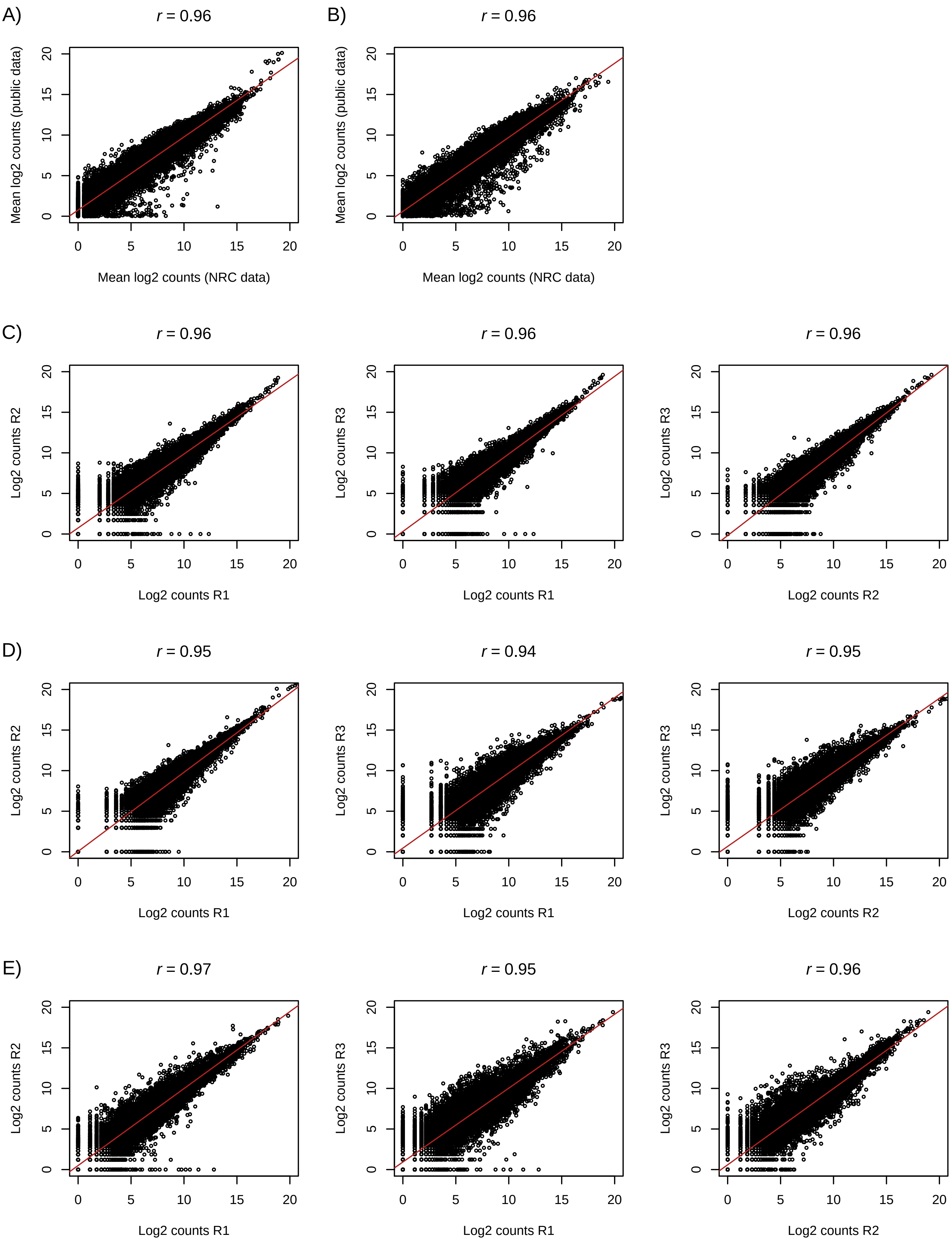
A)
r = 0.96
Mean log2 counts (public data)
Mean log2 counts (NRC data)
B)
r = 0.96
Mean log2 counts (public data)
Mean log2 counts (NRC data)
C)
r = 0.96
Log2 counts R2
Log2 counts R1
r = 0.96
Log2 counts R3
Log2 counts R1
r = 0.96
Log2 counts R3
Log2 counts R2
D)
r = 0.95
Log2 counts R2
Log2 counts R1
r = 0.94
Log2 counts R3
Log2 counts R1
r = 0.95
Log2 counts R3
Log2 counts R2
E)
r = 0.97
Log2 counts R2
Log2 counts R1
r = 0.95
Log2 counts R3
Log2 counts R1
r = 0.96
Log2 counts R3
Log2 counts R2

Supplementary figure 2

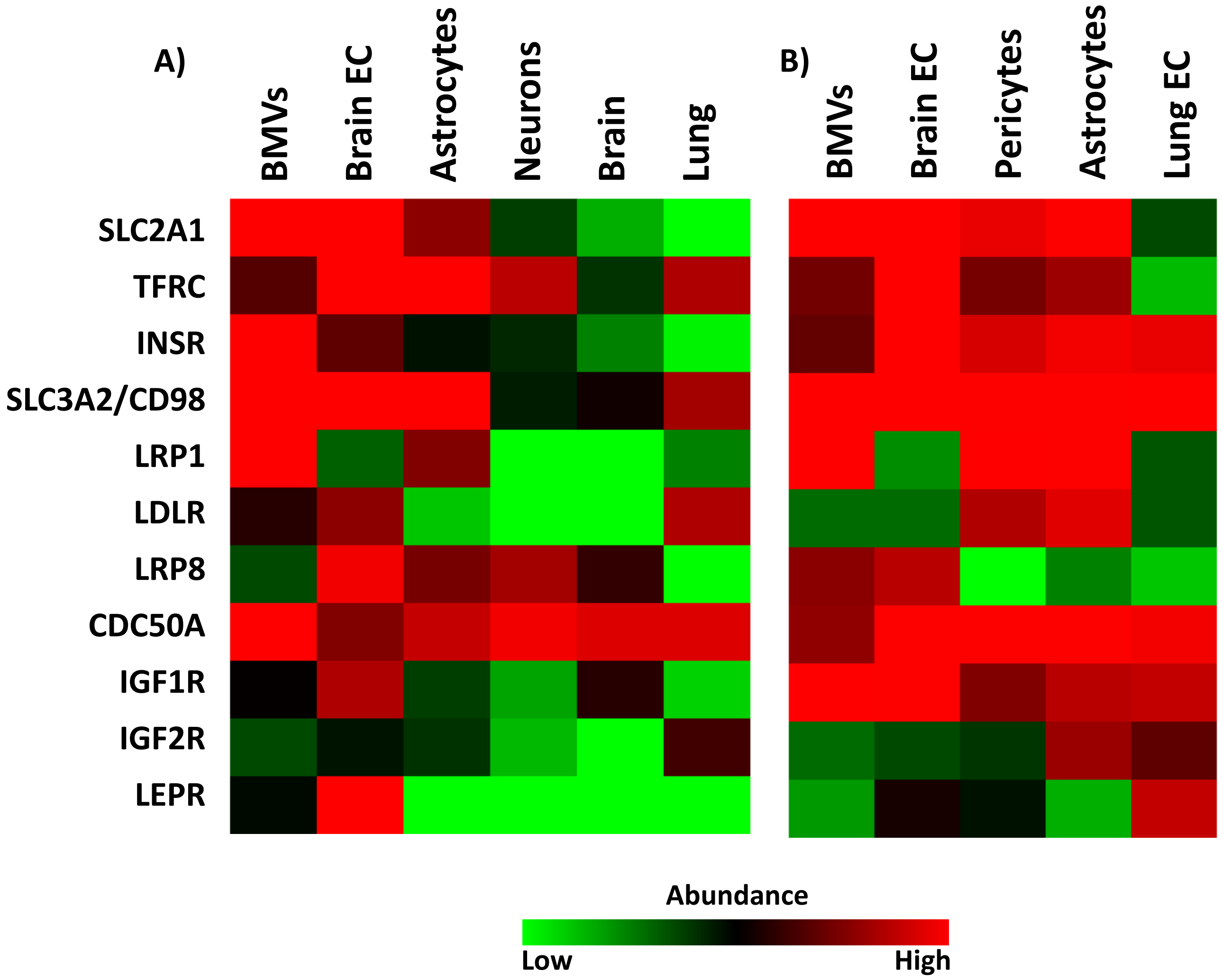

Supplement: Supplementary file 1 — Additional file 1. Additional figures and tables. [file 12987_2020_209_MOESM1_ESM.pdf]
